# Development of Korean Smartphone Addiction Proneness Scale for Youth

**DOI:** 10.1371/journal.pone.0097920

**Published:** 2014-05-21

**Authors:** Dongil Kim, Yunhee Lee, Juyoung Lee, JeeEun Karin Nam, Yeoju Chung

**Affiliations:** 1 Department of Education, Seoul National University, Seoul, South Korea; 2 Department of Education, Korea National University of Education, CheongJu, South Korea; University of Missouri-Kansas City, United States of America

## Abstract

This study developed a Smartphone Addiction Proneness Scale (SAPS) based on the existing internet and cellular phone addiction scales. For the development of this scale, 29 items (1.5 times the final number of items) were initially selected as preliminary items, based on the previous studies on internet/phone addiction as well as the clinical experience of involved experts. The preliminary scale was administered to a nationally representative sample of 795 students in elementary, middle, and high schools across South Korea. Then, final 15 items were selected according to the reliability test results. The final scale consisted of four subdomains: (1) disturbance of adaptive functions, (2) virtual life orientation, (3) withdrawal, and (4) tolerance. The final scale indicated a high reliability with Cronbach's α of .880. Support for the scale's criterion validity has been demonstrated by its relationship to the internet addiction scale, KS-II (r  =  .49). For the analysis of construct validity, we tested the Structural Equation Model. The results showed the four-factor structure to be valid (NFI  =  .943, TLI  =  .902, CFI  =  .902, RMSEA  =  .034). Smartphone addiction is gaining a greater spotlight as possibly a new form of addiction along with internet addiction. The SAPS appears to be a reliable and valid diagnostic scale for screening adolescents who may be at risk of smartphone addiction. Further implications and limitations are discussed.

## Introduction

The propagation of personal computers in the 1990's gave birth to a digital revolution. Personal desktops soon evolved into PMPs, tablet PCs, and smartphones – devices that have become increasingly common in people's lives. In particular, the distribution rate of smartphones is in an upward trend worldwide since 2000 [1]. Such widespread use of smartphones has been named “Smart Revolution,” and has been bringing dramatic changes in people's daily lives. Although smartphone usage has made life more convenient for many people, it has also brought about adverse effects in the realms of psychological well-being, interpersonal relationships, and physical health. For instance, due to easy access to online environment through smartphones, negative consequences of *online disinhibition effect* characterized by lowered behavioral inhibitions [2] [3] are becoming more rampant, particularly in forms of cyber violence.

Today's adolescents are highly receptive of new forms of media such as smartphones [4] as they are the first generation to have grown up surrounded by various forms of high-tech media [5]. This could mean that youths are more susceptible to the adverse effects of smart media than older age groups. In South Korea, youths addicted to smartphone have reached 11.4% of the population, with the top 2.2% facing difficulty living out their everyday lives due to their addiction [6]. Before the spread of smartphones, cell phones took up a huge part of adolescents' lives to the point where some reported experiencing high levels of anxiety when their phone is not always on [4]. Cellular phone addiction and age seem to be inversely proportional, with younger people using their phones more frequently [8], and two times more likely to admit to being a “cellular phone addict” than adults [9]. For adolescents, phone-based communication is an important way to maintain their social relationships [7]. As smartphone addiction is becoming a major issue amongst youths, developing a scale that can estimate the levels and conditions of smartphone addiction among adolescents seems urgent in order to protect them from the addiction's adverse effects.

Because the distribution of smartphones is a relatively recent phenomenon, studies that have defined the unique symptoms of smartphone addiction are rare. The closest concept to smartphone addiction may be cellular phone addiction, which is considered to be a type of behavioral addiction characterized by problems with impulse control. Reported symptoms of cellular phone addiction include withdrawal, tolerance, disturbance of adaptive functions, compulsion, and pathological immersion [12] and abstinence, lack of control and problems derived from the use, and tolerance and interference with other activities [13]. Existing cellular phone addiction scales [47] [48] [49] have been developed based on Young [10]’s Internet Addiction Test (IAT) and Goldberg [11]’s diagnostic criteria for internet addiction.

However, smartphones are different to cellular phones in four major ways. First, smartphone users are more dynamically involved with the device than regular cell phone users. Smartphone users actively engage with the device itself and the contents (applications) simultaneously, and may play a role of producer by creating personalized applications. Since applications allow smartphone users to give immediate, mutual feedback, smartphone users tend to be active, participatory, relational, competent, and productive [15]. Consequently, smartphone usage has been shown to be directly proportional to application usage [14]. Second, smartphones place a greater importance on the sensory features that stimulate users' expressive side [16]. Smartphone's distinctive user interface system, which includes touch screen operation, keyboard arrangements, icons, sensible design, and other components, enables its user to reveal his or her individuality [17]. The importance of the expressive aspect of smartphone applications can also be seen in the fact that users prefer applications that allow multiple users to have fun together and to be socially expressive over applications that can only be enjoyed alone [18]. Third, smartphones provide a convergence of services such as the camera, MP3, GPS, web browsing, calling, e-mail, gaming, and social networking services (SNS) [19] [20] on one portable device. Also called the “handheld Internet,” smartphones' portability allows for real-time and personalized services anywhere which could not be fulfilled on a typical desktop computer. Moreover, smartphone's “Push Service” notifies the users with relevant updates, such as newest emails or Facebook replies, even before the user asks for them [21]. Such personalized services provided by smartphones can be helpful, but may also induce people to overuse their smartphones [22] [23]. Finally, people of different age groups show varying smartphone usage patterns. Teens mainly use their smartphones for the camera, MP3, and other entertainment functions; people in their 20's mainly use SNS; and the people in their 30's and 40's typically manage their schedules, contacts list, e-mail, and other business related functions [24] [25].

Despite smartphones' distinctive characteristics as mentioned above, many of the existing smartphone addiction scales were identical to the cellular phone addiction scale, with the word “cellular phone” simply replaced with “smartphone”. One of the most recent, Casey [26] 's smartphone addiction scale had also extracted items from scales that measure other types of media addiction such as the Mobile Phone Problem Use Scale [27], Internet Addiction Test [10], and Television Addiction Scale [28]. Besides, since cellular phone addiction was also seen as a type of behavioral addiction due to impulse control problems, it was usually comprised with elements from internet addiction.

Therefore, the current study developed the Korean Smartphone Addiction Proneness Scale (SAPS) for Youth by adding items that reflect unique characteristics of smartphones to the Internet Addiction Proneness Scale (IAPS) for Youth [29]. The IAPS is a 20-item scale that has been used to check the level of internet addiction among youth in South Korea since 2007. The SAPS developed through current study will be a useful tool for examining the phenomenon of smartphone overuse among youths, and will ultimately contribute to preventing smartphone addiction.

## Method

### Participants

This study is a secondary data analysis on the national survey data from the National Information Agency of Korea's project on smartphone addiction conducted in 2012 [34]. The researchers of this study had participated in the project as the principal investigator and assistant researchers. Because this project was conducted at a national level, the resulting data was from a large-scale sample that is representative in terms of region, age, and gender. The distributed survey explicitly stated the purpose of the project and notified the participants that they are consenting to participate by filling out the survey. In proportion to the actual population distribution across Korea, 795 elementary, middle, and high school students (461 male and 324 female) had completed the survey. Regional agencies were randomly selected from each of the four areas: Seoul Metropolitan area, Chungcheong/Gangwon area, Honam (including Jeju) area, and Yeongnam area. Many (44.7%) were middle school students, followed by high school students (37.7%) and upper elementary school students (17.6%).

### Measures

#### Demographic Questionnaire

A demographic questionnaire that included items pertaining to student's personal information, extent and nature of smartphone use, and academic performance was included in the survey packet.

#### Smartphone Addiction Proneness Scale Items

Based on the previously developed diagnostic scales and research findings, as well as clinical experiences of numerous specialists, items that theoretically and empirically represent the distinct characteristics of smartphone addiction were selected to comprise the scale. The preliminary scale was composed of twenty-nine items, and each item was scored on a 4-point Likert scale (1  =  strongly disagree, 2  =  disagree, 3  =  agree, 4  =  strongly agree). The twenty-nine preliminary items were structured around four subdomains: disturbance of adaptive functions (9 items), withdrawal (7 items), tolerance (6 items), and virtual life orientation (7 items).

#### Mental Health Problems Scale

To check the validity of the SAPS, a measure that assesses mental health problems related to smartphone addiction was developed. Psychological difficulties that could accompany smartphone addiction include anxiety, depression, impulsiveness, and aggression [50]. Thus, NEO Youth Personality Test [30] items related to these problems (factors) were modified and included in the current scale. The scale consists of 32 items, 8 items for each factor. Items are rated on a 4-point scale (1  =  strongly disagree, 2  =  disagree, 3  =  agree, 4  =  strongly agree). The inter-item consistency for the scale is high with a Cronbach's alpha of .944 overall and .865, .870, .820, .878 for each factor.

#### Internet Addiction Proneness Scale for Youth (KS-II)

To compare smartphone addiction with internet addiction, the 15-item KS-II was used. KS-II developed by the National Information Society Agency [31] has gone through the standardization process in Korea through a nation-wide field survey. KS-II is structured around the four factors: (1) disturbance of adaptive functions, (2) withdrawal, (3) tolerance, and (4) virtual life orientation. Items are rated on a 4-point scale (1  =  strongly disagree, 2  =  disagree, 3  =  agree, 4  =  strongly agree). The inter-item consistency for the scale is high with a Cronbach's alpha of .87.

### Procedure

First, upon reviewing the related scales that were previously developed and examining their theoretical backgrounds, specialists selected items for a preliminary questionnaire. This initial pool had about twice as many items as the final scale. The preliminary scale was administered to students and data were collected. Then, final items were selected according to the reliability test results for each subscale. Finally, the construct validity model for each subdomain was validated on AMOS. A more detailed description of each step of the procedure is as follows.

#### Preliminary Smartphone Addiction Proneness Scale for Youth

A pool of preliminary items for Smartphone Addiction Proneness Scale (SAPS) for youth was developed based on the findings from previous literature on internet addiction, mobile phone addiction, and digital media addiction. Since smartphone is a mobile device that enables internet use, existing internet addiction scales were used for reference. The characteristics of digital media addiction suggested by Young [38] and Greenfield [44] were also reflected in the developed items. Considering that smartphones can be seen as advanced versions of regular mobile phones, existing mobile phone scales [12] [8] were examined as well. Consequently, the subdomains of SAPS came to include disturbance of adaptive functions, withdrawal, tolerance, and virtual life orientation. Finally, experts (education specialists, psychiatrists) created 29 preliminary items that reflect these four subdomains of smartphone addiction.

#### Scale Administration

The SAPS was distributed in randomly selected elementary, middle, and high schools so that participants can be selected in proportion to the actual population distribution across Korea.

#### Item Selection through Reliability Analysis

Reliability analyses on the 29 preliminary items were conducted by subdomain. A total of 15 items that seem adequate were selected. Finally, the Cronbach's alpha for the final scale with 15 items was calculated.

#### Construct Validity Model for Each Subdomain

To confirm the construct validity of SAPS, the construct validity model for each subdomain was validated on AMOS.

## Results

### Selection of Final Items through Reliability Analyses on Subdomains

From the original 29 items, items that seemed unsuitable for each subdomain were deleted or revised based on the results of reliability analyses. To verify the reliability of the items in each subdomain, Cronbach's alphas were examined. The items that lowered the overall reliability of the subdomain if deleted as well as the items with the highest reliability were selected for the final scale. Also, to detect careless or inconsistent responders, reverse-coded items with high reliability were included. Table 1 below displays the reliability results of each subdomain, and Table 2 displays the final 15 items selected.

**Table 1:** Selection of Final Items through Reliability Analysis on Subscales.

Subdomain	Item No.	Average	SD	Scale Mean if Item Deleted	Scale Variance if Item Deleted	Corrected Item-Total Correlation	Cronbach's a if Item Deleted
**Disturbance of Adaptive Functions**	SP 21	1.6321	.75269	14.7310	17.868	.714	.796
	SP 20	1.7524	.82749	14.6107	17.792	.645	.802
	SP 17	1.6869	.72636	14.6762	18.336	.661	.802
	SP 19	1.7536	.82134	14.6095	17.864	.639	.803
	SP 15	1.5964	.67845	14.7667	18.644	.661	.804
	SP 18	1.6179	.77531	14.7452	18.471	.586	.809
	SP 14	2.0274	.83171	14.3357	18.865	.474	.822
	SP 22	2.1452	.93806	14.2179	18.850	.399	.833
	Re_SP 16	2.1512	.95163	14.2119	20.146	.225	.854
**Withdrawal**	SP 2	1.8355	.77344	12.2062	9.613	.711	.706
	SP 1	2.0203	.83384	12.0215	9.637	.635	.719
	SP 4	1.6019	.68832	12.4398	10.512	.590	.734
	SP 7	1.6961	.73888	12.3456	10.327	.579	.734
	SP 5	2.2872	.89401	11.7545	10.458	.409	.769
	Re_SP 3	2.2586	.86903	11.7831	10.776	.366	.777
	Re_SP 6	2.3421	.82966	11.6996	11.334	.286	.791
**Tolerance**	SP 11	2.1085	.89358	10.5542	8.646	.691	.703
	SP 10	1.9583	.82874	10.7044	9.275	.617	.725
	SP 8	1.7366	.84812	10.9261	9.500	.546	.742
	SP 13	2.3576	.92223	10.3051	9.611	.457	.765
	Re_SP 12	2.4720	.89622	10.1907	9.751	.450	.766
	Re_SP 9	2.0298	.85056	10.6329	10.101	.416	.773
**Virtual life orientation**	SP 24	1.5317	.65692	10.9030	8.515	.594	.610
	SP 23	1.4754	.65540	10.9593	8.579	.577	.614
	SP 29	1.5689	.71870	10.8659	8.466	.535	.619
	SP 26	1.6335	.69760	10.8012	8.646	.509	.627
	SP 28	1.6228	.71484	10.8120	8.738	.467	.636
	Re_SP 27	2.1329	.99172	10.3018	8.520	.297	.688
	Re_SP 25	2.4695	1.12096	9.9653	9.261	.103	.765

**Table 2:** Final Items.

Subdomain	Items	No.
**Disturbance of Adaptive Functions**	My school grades dropped due to excessive smartphone use.	1
	I have a hard time doing what I have planned (study, do homework, or go to afterschool classes) due to using smartphone.	5
	People frequently comment on my excessive smartphone use.	9
	Family or friends complain that I use my smartphone too much.	12
	My smartphone does not distract me from my studies.	13*
**Virtual Life Orientation**	Using a smartphone is more enjoyable than spending time with family or friends.	2
	When I cannot use a smartphone, I feel like I have lost the entire world.	6
**Withdrawal**	It would be painful if I am not allowed to use a smartphone.	3
	I get restless and nervous when I am without a smartphone.	7
	I am not anxious even when I am without a smartphone.	10*
	I panic when I cannot use my smartphone.	14
**Tolerance**	I try cutting my smartphone usage time, but I fail.	4
	I can control my smartphone usage time.	8*
	Even when I think I should stop, I continue to use my smartphone too much.	11
	Spending a lot of time on my smartphone has become a habit.	15

* Reverse-coded items.

### Reliability

The reliability of SAPS was verified with a Cronbach's alpha of 0.88.

### Validity

#### Criterion Validity Analysis

To confirm the criterion validity of SAPS, the scores from SAPS and Mental Health Problems Scale were compared. Table 3 shows the Pearson correlation results of the two scales. As a result, the correlation coefficient came out to be 0.43. Furthermore, the correlations among the subscales of SAPS and the Mental Health Problems Scale were all in the 0.49∼0.67 range, confirming a certain degree of correlation.

**Table 3:** Correlation analysis between SAPS and the Mental Health Problems Scale.

	1	2	3	4	5
Total Score (SAPS)	.85**	.70**	.81**	.87**	.43**
Disturbance of Adaptive Functions		.55**	.49**	.67**	.36**
Virtual Life Orientation			.55**	.47**	.34**
Withdrawal				.63**	.37**
Tolerance					.36**

1, Disturbance of Adaptive Functions; 2, Virtual Life Orientation; 3, Withdrawal; 4, Tolerance; 5, Mental Health Problems Scale.

The correlation between SAPS and the KS-II were analyzed; Table 4 shows the results of Pearson's correlation analysis. The correlation coefficient of 0.49 showed that if the score on SAPS was high, the KS-II score was probably high as well. Additionally, the correlations among the subscales of the KS-II and SAPS were between 0.12 and 0.51, again showing a certain degree of correlation.

**Table 4:** Correlation analyses between SAPS and KS-II.

		SAPS				KS-II				
		1	2	3	4	5	1	2	3	4
**SAPS**	Total Score	.84**	.78**	.83**	.86**	.49**	.48**	.34**	.21**	.32**
	Disturbance of Adaptive Functions		.53**	.50**	.68**	.49**	.51**	.31**	.16**	.38**
	Virtual Life Orientation			.51**	.47**	.26**	.25**	.30**	.22**	.23**
	Withdrawal				.59**	.42**	.43**	.30**	.22**	.23**
	Tolerance					.53**	.49**	.27**	.12**	.41**
**KS-II**	Total Score						.85**	.68**	.57**	.81**
	Disturbance of Adaptive Functions							.53**	.25**	.68**
	Virtual Life Orientation								.19**	.50**
	Withdrawal									.15**

1, Disturbance of Adaptive Functions; 2, Virtual Life Orientation; 3, Withdrawal; 4, Tolerance; 5, Total Score.

#### Construct Validity Analysis

**Figure 1:**
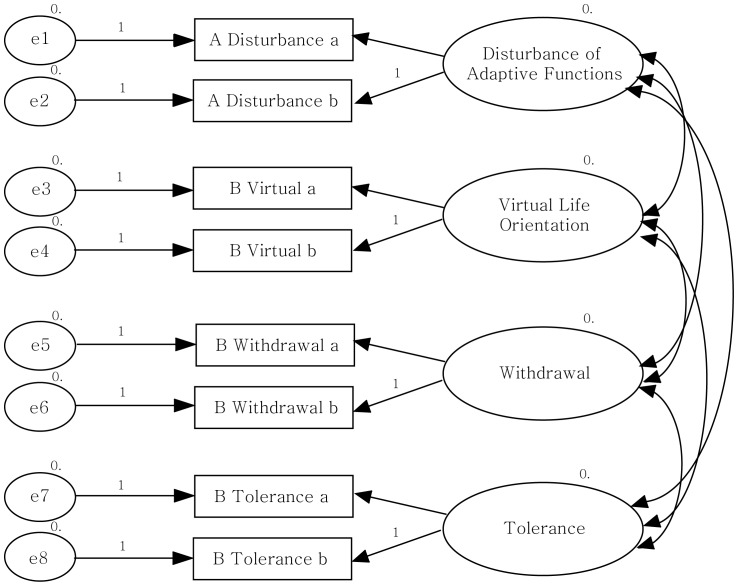
The factor structure of SAPS. The structural model of the four subdomains of smartphone addiction (disturbance of adaptive functions, virtual life orientation, withdrawal, and tolerance) and their pertinent items appeared valid.

Confirmatory factor analysis was conducted using AMOS 7.0 to confirm the factor structure of SAPS. For this, the factor structure model was set as follows (Figure 1).

First, the model fit indices NFI, TLI, CFI, and RMSEA were .943, .902, .962, and .034 respectively, showing that the pertinent model was well suited to the data. Therefore, the structural model of the four subdomains of smartphone addiction (disturbance of adaptive functions, virtual life orientation, withdrawal, and tolerance) and their pertinent items appeared valid.

Also, to figure out how comprehensively each item explains the related factors, the regression coefficient of each observable variable and its degree of statistical significance were examined. In all observable variables except for “virtual life orientation,” the standardized coefficients were greater than .5 on average, which was statistically significant (*p*<.001). Table 5 displays these statistics.

**Table 5:** Regression coefficients of observable variables regarding each factor.

			Unstandardized Coefficients	Standardized Coefficients	S.E.	C.R.	P
A disturbance b	←	1	1.000	.691			
A disturbance a	←	1	1.052	.920	.102	10.349	***
B virtual b	←	2	1.000	.874			
B virtual a	←	2	.851	.651	.102	8.345	***
B withdrawal b	←	3	1.000	.675			
B withdrawal a	←	3	1.428	.816	.166	8.604	***
B tolerance b	←	4	1.000	.819			
B tolerance a	←	4	.814	.657	.087	9.358	***

1, Disturbance of Adaptive Functions; 2, Virtual Life Orientation; 3, Withdrawal; 4, Tolerance; 5, Mental Health Problems Scale.

***p < 0.001.

## Discussion

As a part of the National Information Agency of Korea's project on youth smartphone addiction conducted in 2012 [34], this study sought to lay foundations for prevention/intervention efforts for youth smartphone addiction. Specifically, the study developed a brief 15-item smartphone addiction proneness scale that could be used in nation-wide data collection efforts. The developers paid a particular attention to the simplicity of scale items and the ease of use in scale administration in order to facilitate actual utilization.

Cronbach's alpha of the final SAPS was .880, demonstrating the scale to be reliable. Existing internet addiction or smartphone scales have also been reported to be reliable with Cronbach's alphas of above .7. However, it may be unwise to trust their reliability values because their data collection process was not standardized or their sample size was small. For instance, Beard and Wolf [37] attempted to improve on Young [38] 's Diagnostic Criteria for Internet Addiction, but their scale development process was not standardized. Widyanto and McMurren [39], on the other hand, did follow a standardized procedure for scale development, but failed to collect enough data (n = 86). Moreover, they collected data online, which could mean that their data collection was biased. Similar limitations are present among existing smartphone addiction scales as well. Kwon et al. [36] had developed a scale based on the K-scale items and the smart device characteristics, and reported the scale to have a Cronbach's alpha of .91. However, it must be noted that their data collection took place at two schools located in one particular region in Korea, thus raising questions about their scale's reliability value. Thus, SAPS of this study can be considered more reliable compared to existing scales as it was developed based on the data collected from 795 students across Korea in proportion to the actual population distribution of the nation.

SAPS appeared to be validly structured around four subdomains (adaptive functions, withdrawal, tolerance, and virtual life orientation) of smartphone addiction. In order to decide on the scale's subdomains, previous research with a particular attention to studies on internet addiction scales and the diagnostic criteria for other behavioral addictions were examined. Factors that commonly appear among these studies as well as factors that reflect the characteristics of smartphones were included. A confirmatory factor analysis was conducted using AMOS 7.0 to verify the construct validity of the scale. Finally, the correlations between SAPS and KS-II (an internet addiction scale) as well as between SAPS and the Mental Health Problems Scale were checked in order to confirm the criterion validity of SAPS.

Internet addiction scales developed and validated in various countries vary in their factor structures. Canan et al. [40] developed an internet addiction scale for Turkish adolescents and found that its items were grouped as one factor. Similarly, Khazaal et al. [41] developed an internet addiction scale for French adults and found that its items were grouped as one single factor. However, other studies have reported that their internet addiction scale items were grouped into various factors, such as obsession, neglect, and control disorder [42] [43]. Korea's most commonly used K-scale is also composed of many factors, such as adaptive functions, withdrawal, tolerance, and virtual life orientation. As such, scholars seem to disagree on the subdomains of internet addiction scales, implying that the factor structure of internet addiction scales may not be quite stable.

The limitations of this study and suggestions for future studies are as follows.

First, ‘tolerance,’ a subdomain of SAPS as well as the internet addiction scales, is not a core factor of addiction according to Charlton and Danforth [45]. In other words, using the internet for many hours itself cannot be a criterion for addiction until such behavior results in negative consequences [35]. Since smartphones are devices that people carry around and use everywhere, tolerance may be unfit as the core factor of smartphone addiction. This calls for additional nationwide survey and data analyses on this topic. Moreover, the validation of the scale could be improved by, for example, administering the scale to populations of addicted and non-addicted youth to examine the scale's discriminant validity.

Next, SAPS for youth can widely used in smartphone addiction research that is gaining momentum these days. Today's digital media devices have been rapidly developed from PC-based forms to smartphones and various tablet PCs. In other words, existing media and recent media are going through competition as well as substitution process. Since youths these days are considered as digital natives [46] who actively accept and use the most up-to-date media [32], investigating the possible side effects of their media use on their mental health seems urgent. Excessive use of digital media can bring negative consequences in physical, psychological, and social aspects of adolescents' lives, and may even trigger delinquent behaviors. For instance, Kross et al. [33] found that Facebook use is not helpful for social interaction and is associated with low levels of subjective psychological well-being. Therefore, research into symptoms of smartphone addiction as well as effects of smartphone addiction on adolescent mental health is necessary, and SAPS can be well utilized in such endeavor.
